# Improvement of Raw Milk Cheese Hygiene through the Selection of Starter and Non-Starter Lactic Acid Bacteria: The Successful Case of PDO Pecorino Siciliano Cheese

**DOI:** 10.3390/ijerph18041834

**Published:** 2021-02-13

**Authors:** Raimondo Gaglio, Massimo Todaro, Luca Settanni

**Affiliations:** Department of Agricultural, Food and Forestry Science, University of Palermo, Viale delle Scienze 4, 90128 Palermo, Italy; raimondo.gaglio@unipa.it (R.G.); massimo.todaro@unipa.it (M.T.)

**Keywords:** lactic acid bacteria, microbial variability, starter selection, traditional cheese, bacterial stabilization

## Abstract

This review article focuses on the technological aspects and microbiological critical points of pressed-cooked cheeses processed from raw ewe’s milk without the inoculation of starter cultures, in particular “Pecorino” cheese typology produced in Italy. After showing the composition of the biofilms adhering to the surface of the traditional dairy equipment (mainly wooden vat used to collect milk) and the microbiological characteristics of PDO Pecorino Siciliano cheese manufactured throughout Sicily, this cheese is taken as a case study to develop a strategy to improve its hygienic and safety characteristics. Basically, the natural lactic acid bacterial populations of fresh and ripened cheeses were characterized to select an autochthonous starter and non-starter cultures to stabilize the microbial community of PDO Pecorino Siciliano cheese. These bacteria were applied at a small scale level to prove their in situ efficacy, and finally introduced within the consortium for protection and promotion of this cheese to disseminate their performances to all dairy factories. The innovation in PDO Pecorino Siciliano cheese production was proven to be respectful of the traditional protocol, the final cheeses preserved their typicality, and the general cheese safety was improved. An overview of the future research prospects is also reported.

## 1. Introduction

The origin of cheese is particularly ancient; it is dated back to 8000 years ago when this dairy fermented product was already produced in the Middle-East [1]. Italy is characterized by a long history of cheese production. In Sicily, which represents the largest island in the Mediterranean sea, the production of cheese began with the Phoenician community [2], and the discovery of several archaeological finds indicated that the dairy activity was routinely conducted in the island in the Eneolithic age [3].

Generally, all hard cheeses made from ewes’ milk produced in Italy are referred to as “Pecorino” cheeses. The term Pecorino is an adjective that comes from “pecora,” the Italian translation of ewe. The wider tradition of raw ewe’s milk transformation is concentrated in the southern regions [4,5]. Indeed, Pecorino cheese is produced almost throughout Italy, with names strictly indicating the geographical origin like Pecorino Romano, Pecorino Siciliano, Pecorino Sardo, and Pecorino Toscano among the most known products [6]. All these cheeses are considered typical products since they are transformed by the application of traditional production processes in given geographical areas [7]. Most of them enjoy the “protected designation of origin” (PDO) status, a recognition of quality conferred by the European Community to protect some agricultural products and foodstuffs produced in a specific area and following their production protocols [8].

PDO Pecorino Siciliano cheese is produced from raw ewe’s milk without the inoculation of starter cultures in milk using traditional wooden equipment. In these conditions, the microbiota responsible for the acidification of curd and maturation of cheese originates from the raw milk, the equipment, the animal rennet, and, in general, the transformation (dairy) environment [9,10]. The microorganisms necessary to transform milk into cheese are lactic acid bacteria (LAB): Those acting during the first hours/days of productions are starter species, which have to generate a high amount of lactic acid basically through the fermentation of lactose, while the species acting during ripening, important to develop the final organoleptic notes, are indicated as non-starter LAB [11]. Both groups of LAB contribute to controlling the development of undesired microorganisms, but their activities and the stressing physicochemical conditions of the mature cheeses, mainly represented by low pH, high concentrations of organic acids, low water activity, and moderate sodium chloride content [12], might not be enough to eliminate the presence of spoilage and opportunistic pathogen microorganisms from Pecorino Siciliano cheese [6].

Todaro et al. [6] isolated and identified *Citrobacter freundii* and *Stenotrophomonas maltophilia* among the potential pathogenic bacteria and *Pseudomonas putida* within the spoilage agents at consistent levels, together with several non-starter LAB, in some PDO Pecorino Siciliano cheeses. This paper reviews the strategy planned, and the works performed to improve the hygienic and safety aspects of PDO Pecorino Siciliano cheese based on the isolation, characterization, and selection of starter and non-starter LAB, as well as their application at the laboratory, small scale, and large scale level.

## 2. PDO Pecorino Siciliano Cheese Technology

**Figure 1:**
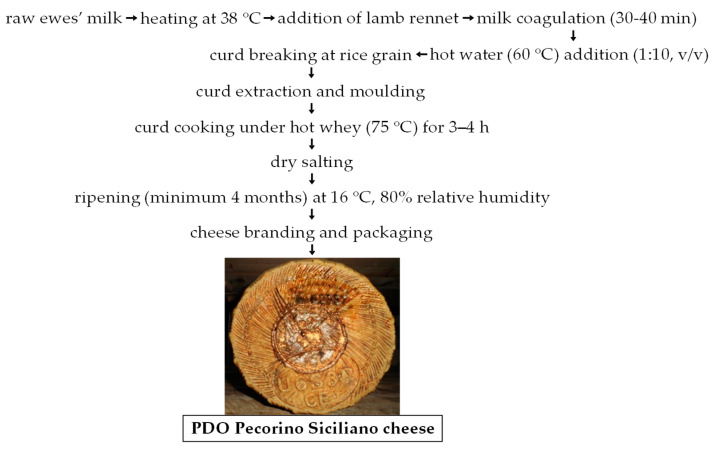
Main steps of protected designation of origin (PDO) Pecorino Siciliano cheese production. Adapted from Settanni et al. [16].

PDO Pecorino Siciliano is a semi-hard cheese produced following a century-old protocol from raw ewe’s milk collected into wooden vats where it is transformed, adding animal rennet in a paste, dissolved into warm water before addition, without LAB starter inoculums. The production area of this PDO cheese is quite wide, because it is represented by the entire Sicily region (South Italy) with its 25,711 km^2^. The protocol of production was regulated in 1955 [13] making PDO Pecorino Siciliano cheese probably one of the oldest European cheeses whose production has been regulated [14]. PDO Pecorino Siciliano cheese is counted among the pressed-cooked cheeses [15], and the transformation process is graphically reported in Figure 1.

In particular, the milk is preheated at a temperature of 38 °C, even though in the practice of the dairy factories, a temperature of 35–37 °C is often registered. The curd is then broken by a wooden stick beater (called “rotula” in the local dialect) until reaching pieces of rice grain dimensions, and the curd is pressed into rattan baskets in order to facilitate the draining of the whey. These containers also provide the final shape to the cheeses, which are cooked under hot (about 75 °C) deproteinized whey, namely “scotta whey” (residual from Ricotta cheese production [17]) for about 3–4 h just after molding. Curdling and cooking are performed into the same wooden vat, called “tina” in the local dialect, where raw milk was collected. The traditional wooden equipment used for PDO Pecorino Siciliano cheese production is mainly made of chestnut or Douglas fir, genus *Pseudotsuga*, wood [18], even though recent attempts to introduce other local wood typologies of the Sicilian forestry resources showed interesting results during raw ewes’ milk cheese production [19,20].

## 3. Role of the Wooden Vat during PDO Pecorino Siciliano Cheese Production

The European Regulation (EC) no. 1935/2004 refers to the materials in contact with foods [21]. Basically, any material must not transfer its constituents to foods. Even though the measures regarding plastics, active and intelligent materials, epoxy derivatives, regenerated cellulose, and ceramics have been harmonized and adopted at the European level, there are no specific indications for wood as a food contact material. Thus, member countries legislate at different levels [22]. E.g., in France and Italy (mainly in the Sicily region), wooden equipment is used for dairy purposes thanks to the EC no. 2074/2005, which allows derogation from the EC no. 852/2004 for foods with traditional characteristics “as regards the type of materials of which the instruments and the equipment used specifically for the preparation, packaging, and wrapping of these products are made” [23].

**Figure 2:**
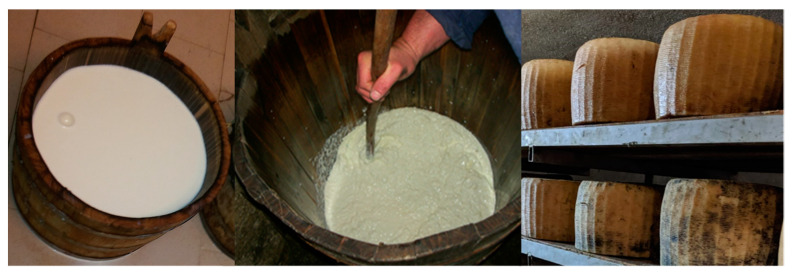
Contact of raw milk and ripened cheeses with wooden equipment during the production of PDO Pecorino Siciliano cheeses.

Traditional Sicilian cheeses are in contact with wood during the entire production process, from milking until ripening. Figure 2 reports the main steps of production of PDO Pecorino Siciliano cheese, highlighting the constant contact with wooden equipment.

**Figure 3:**
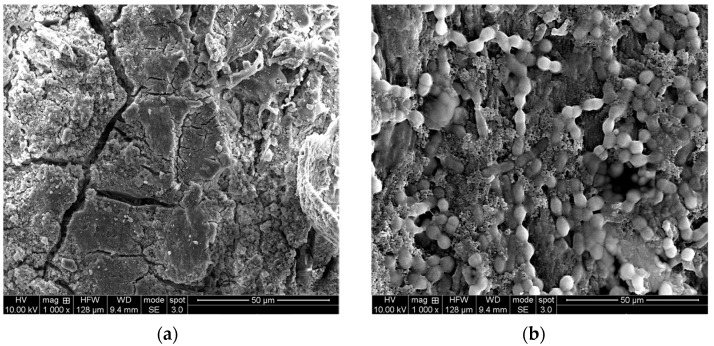
Scanning electron microscopy observations of wooden vat surface. (**a**) Before microbial activation; (**b**) after contact with scotta whey.

Among the wooden equipment generally used to produce the typical Sicilian cheeses, the vat used to collect the bulk milk for curdling assumes the most important microbiological role because it acts as a reservoir of starter LAB necessary to acidify the curd [18,24,25,26]. The wooden vat is also important to provide the non-starter LAB responsible for the desirable changes occurring during cheese ripening [27,28,29]. Both starter and non-starter LAB are transferred from the wooden vat to the milk during the first minutes of contact [18,24,30] thanks to the biofilms formed on its internal surface [19,31]. A given biofilm is an aggregate of microorganisms embedded into their exopolysaccharides (EPS) adhering to a solid surface [32,33]. The ultrastructure of wood surfaces is porous (Figure 3a), facilitating the absorption and trapping of bacteria, which may develop a complex biofilm (Figure 3b).

**Figure 4:**
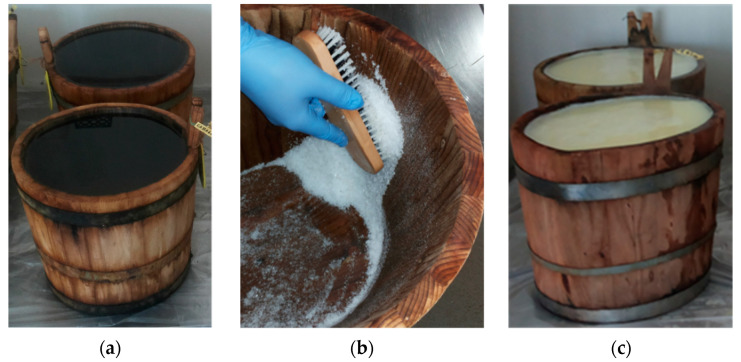
Bacterial activation of wooden vat surfaces with dairy lactic acid bacteria (LAB): (**a**) Tannin removal with hot water for 30 days; (**b**) coarse salt brushing; (**c**) Scotta whey contact for 7 days.

In order to allow the development of the desired LAB onto the internal surfaces of the wooden vats (early bacterial biofilm formation, Figure 4), the new vats, just after their construction, undergo the first treatment with hot water (80 °C) for approximately 30 consecutive days (Figure 4a). This treatment is aimed at removing the tannin components. Subsequently, the internal surfaces of the vats are vigorously brushed with coarse salt (Figure 4b) and filled in with hot water once again to remove the salt. After this surface preparation protocol is applied, the biofilms formed through the daily contact with hot (approximately 70 °C) scotta whey (Figure 4c) for almost seven consecutive days [19].

**Figure 5:**
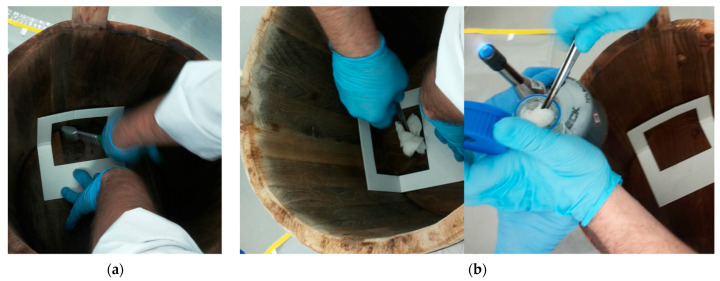
Wooden vat biofilm collection: (**a**) Brushing; (**b**) collection by gauze.

The wooden vat biofilms associated with the production of PDO Pecorino Siciliano cheeses are often the same used for the production of PDO Vastedda della valle del Belìce cheese, which was thoroughly investigated by some authors [18,34]. To this purpose, it is important to note that most of LAB were identified as *Lactobacillus*, but since 2020 the nomenclature of lactobacilli underwent a consistent revision by Zheng et al. [35], and the bacteria previously grouped into the genus *Lactobacillus* are currently split into 23 different genera. However, in order to better understand the results of the research of Scatassa et al. [18,34], as well as those of the other research objectives of this review article, LAB whose nomenclature was revised, will be reported with the current names and the old names between brackets at the first citation. The collection of the wooden vat biofilms occurred by the brushing technique reported by Didienne et al. [30]. Briefly, the vat surfaces were sampled, positioning UV-treated paper squares halfway on the bottom and up the side of the vat (Figure 5).

The biofilms were subjected to plate counts, and the colonies showing different morphological appearances were isolated, purified, and identified by 16S rRNA gene sequencing as belonging to several LAB genera [18,34]. In particular, the species identified were: *Enterococcus durans*, *Enterococcus faecium*, *Enterococcus faecalis*, *Enterococcus hirae*, *Streptococcus lutetiensis*, *Streptococcus gallolyticus* subsp. *macedonicus*, *Streptococcus thermophilus*, *Pediococcus acidilactici*, *Lactococcus lactis*, *Levilactobacillus brevis* (formerly *Lactobacillus brevis*), *Limosilactobacillus fermentum* (formerly *Lactobacillus fermentum*), *Lacticaseibacillus casei* (formerly *Lactobacillus casei*), *Lactobacillus delbrueckii*, and *Lacticaseibacillus rhamnosus* (formerly *Lactobacillus rhamnosus*).

In light of the Commission Regulation (EC) No 2073/2005 [36], Scatassa et al. [18] also applied the microbiological criteria for foodstuffs to the wooden vats used to transform milk into cheese. To this purpose, the presence of *Listeria monocytogenes* and *Salmonella* spp. was investigated as food safety criteria, while that of *Escherichia coli* and coagulase-positive staphylococci as process hygiene criteria. The investigation results showed that *Ls. monocytogenes* and *Salmonella* spp. were not detected, coagulase-positive staphylococci were below the detection limit, while *Es. coli* was found only in one vat out of the five objects of study. Some authors stated that the absence of these pathogenic species onto the surface of the wooden vats is due to their inability to adhere or to survive in the microbial biofilms for a combination of stress conditions such as the production of organic acids, the competition for nutrients, and the generation of antimicrobial compounds, mainly bacteriocins, by LAB and also to the high temperatures applied for cheese cooking [26,37]. To this purpose, Scatassa et al. [18,34] tested all isolates through the general assays applied to evaluate the bacteriocin-like inhibitory substance production [38], finding that a consistent percentage of vat LAB inhibited some indicator strains, including *Ls. monocytogenes* (strain ATCC 19114). These results provided evidence that the wooden vat biofilms contribute actively to the microbial safety of the traditional cheeses.

## 4. Microbial Ecology of PDO Pecorino Siciliano Cheese

Even though PDO Pecorino Siciliano cheese has been produced for centuries, the first documentation regarding the microbial ecology of similar Pecorino Siciliano cheeses was published in 2006 by Vernile et al. [39]. Those authors characterized only the levels of LAB in cheese produced in different seasons, showing that after three months of ripening, mesophilic rods and cocci were approximately at the same level, which was estimated at about 10^7^ cfu/g. Two years later, the same authors [40] reported the genotypic characterization of those LAB showing that the species associated with the ripening of PDO Pecorino Siciliano cheese were *E. faecium*, *E. faecalis*, *Lacticaseibacillus paracasei* (formerly *Lactobacillus paracasei*), *Lactiplantibacillus plantarum* (formerly *Lactobacillus plantarum*), *Lactiplantibacillus pentosus* (formerly *Lactobacillus pentosus*), *Lt. rhamnosus*, *Latilactobacillus curvatus* (formerly *Lactobacillus curvatus*) and leuconostocs. Todaro et al. [6] evaluated the microbiological characteristics of PDO Pecorino Siciliano cheeses of different weights subjected to two different salting technologies (dry salting and a combined dry-brine salting). That work showed that LAB and the undesired spoilage and potentially pathogenic bacteria (pseudomonads and members of *Enterobacteriaceae* family) were almost at the same levels (10^5^ cfu/g) after five months of ripening. LAB populations included *E. durans*, *E. faecium*, *E. faecalis*, *Lactococcus garvieae*, *L. brevis*, *P. acidilactici*, *Pediococcus pentosaceus*, *S. gallolyticus* subsp. *macedonicus*, and *Streptococcus infantarius*. Randazzo et al. [41] revealed the presence of *Lc. lactis*, *S. thermophilus*, *Streptococcus bovis*, *E. faecalis,* and *Leuconostoc mesenteroides* in artisanal Pecorino Siciliano cheeses through the application of a combined culture-dependent and–independent approach. Caggia et al. [42] specifically performed the isolation of non-starter LAB from Pecorino Siciliano cheeses in order to collect a group of LAB to be screened for probiotic features identifying several *Lt. rhamnosus* and *Lt. paracasei*.

The species associated with the wooden equipment and those identified in PDO Pecorino Siciliano cheeses included mesophilic (*Lc. lactis*) and thermophilic (*S. thermophilus*) starter LAB. In general, the thermophilic species are associated with the cheese core [43], where the temperature reached during cooking is maintained for a longer time than at the under rind [44]. However, the majority of the species identified from the wooden vats and ripened PDO Pecorino Siciliano cheeses were allotted into the non-starter LAB group. Among these, in the last years, *S. gallolyticus* subsp. *macedonicus* and *Lc. garvieae* represent adjunct cultures inoculated during different Italian cheese productions [45,46]. Enterococci are responsible for several typicality notes of cheese [47] but may carry antibiotic resistance genes and virulence factors [48]. These bacteria, besides being naturally present in raw milk [49,50] and in the wooden vat biofilms [18,34], have also been isolated from the animal rennet pastes used in traditional Sicilian cheese production [51], indicating that the presence of enterococci in the final cheeses might have different origins. Once their harmlessness is ascertained [48], enterococci can be selected as secondary adjunct cultures also for their contribution to extending cheese shelf life due to the bacteriocin production [47,52]. With the aim of investigating the antibiotic resistance distribution among Pecorino Siciliano cheese enterococci, Russo et al. [53] isolated several strains belonging to the species *E. faecium*. The authors detected a high resistance of the strains towards rifampicin, erythromycin, and ampicillin and also found that several strains exhibited multidrug-resistant phenotypes highlighting concerns regarding the spread of antibiotic resistance by dairy enterococci.

Todaro et al. [6] focused the attention on the isolation and identification of the undesirable bacteria of PDO Pecorino Siciliano cheeses, finding out that this community was composed of *Pseudomonas putida*, *Pseudomonas vronovensis*, *C. freundii*, *Enterobacter* spp., *Es. coli*, *Klebsiella oxytoca*, *Serratia grimesii*, and *Sn. maltophilia*. Although the presence of the potentially pathogenic bacteria *Enterobacter* spp., *K. oxytoca*, *C. freundii,* and *Es. coli* was documented for Italian raw milk Pecorino cheese typology during the first month of ripening [54], the surprising finding of the work of Todaro et al. [6] was that these species were still viable in cheeses ripened for five months. Members of *Enterobacteriaceae* family at high levels have also been reported for raw ewes’ milk cheeses produced in Greece and Portugal [55,56] and their main implication with human health is basically related to the production of biogenic amines [57]. A rapid decline of *K. oxytoca* detected in curd was reported during the ripening of Serra ewe’s cheese [58], and this phenomenon is important to reduce the risk of hemorrhagic colitis caused by this bacterium [59]. Even though *C. freundii* is considered as a low virulence microorganism, it could be responsible for several infections, including pneumonia, diarrhea, and septicemia [60]. *Escherichia coli* is naturally present in the intestine of warm-blooded animals and represents an indicator of fecal contamination, but it is also implicated in gastrointestinal diseases [61].

Regarding spoilage bacteria, *Pseudomonas* spp. are generally isolated from raw ewes’ milk cheeses with a limited ripening time [62]. Generally, pseudomonads are present at high cell densities when pH is high [63]; thus, a rapid pH drop is necessary to hamper their development. Fresh Pecorino Siciliano “primosale” cheeses, namely “primosale”, were specifically investigated at a retail sale by Giammanco et al. [64] for the presence of *Staphylococcus aureus* and *Es. coli* finding out that a consistent percentage of the samples analyzed were positive for these two species. However, the study evidenced that those results depended on post-production contamination, in particular, the retail sale conditions may have played a key role in the development of *St. aureus* and *Es. coli*. Furthermore, Cardamone et al. [65] inoculated the four main dairy pathogenic species (*Es. coli* O157, *Ls. monocytogenes*, *Salmonella* Enteritidis, and *St. aureus*) at the production stage of Pecorino Siciliano cheese and evaluated their behavior during ripening. The results of the investigation indicated that PDO Pecorino Siciliano cheese production conditions determined a consistent decrease in the growth of all four bacteria inoculated.

PDO Pecorino Siciliano cheese was also specifically investigated for the presence of biogenic amines with the aim of correlating these compounds with cheese microbiology [66]. Indeed, biogenic amines are toxic compounds generated through the microbial decarboxylation of amino acids [67]. Only histamine was detected at a high concentration in PDO Pecorino Siciliano cheese. Guarcello et al. [68] selected some cheese LAB that did not carry amino acid decarboxylase genes and found that some of them even showed a biogenic amine degrading ability determining the selection of LAB starters useful to prevent the accumulation of these toxic compounds in experimental cheeses.

In light of the several microorganisms implicated in human pathogenicity and shelf-life issues isolated from ripened Pecorino cheeses, the selection and application of starter strains are of paramount importance to obtain safe and stable cheeses.

## 5. Improvement of PDO Pecorino Siciliano Cheese Hygiene through Selected LAB Addition

Although the wooden vats used to produce PDO Pecorino Siciliano cheeses were proven to be safe systems for milk transformation, the presence of consistent numbers of spoilage/pathogenic bacteria in the final cheeses determined an alert on the entire production system of this PDO cheese. The sole wooden vat LAB biofilms are unable to sanitize the microbiological conditions of raw ewes’ milk, which is often very close to the European limit (<500,000 cfu/mL) for the “good microbiological quality” of raw ewes’ milk to be processed into cheese when it is not thermally treated [69]. Thus, a revision of the production protocol of PDO Pecorino Siciliano cheese based on the addition of selected autochthonous starter and non-starter LAB was suggested to improve the hygienic conditions of the final cheeses.

According to the classification of Mucchetti and Neviani [70], based on the treatment of milk and the type of selected starters added, the Italian cheeses can be produced from pasteurized milk inoculated with selected starters, pasteurized milk inoculated with natural starters, thermal treated milk inoculated with natural starters, raw milk inoculated with selected starters, raw milk inoculated with natural starters, and raw milk without starter addition. PDO Pecorino Siciliano cheese belongs to the last cheese typology, which deserves major microbiological attention due to the survival of the raw milk bacteria during cheese production [71], even though vat LAB exerts antagonistic measures [26,37]. From this perspective, PDO Pecorino Siciliano cheese represented a case study to convert the transformation process from raw milk without starters to a production performed with raw milk added with natural starters.

**Figure 6:**
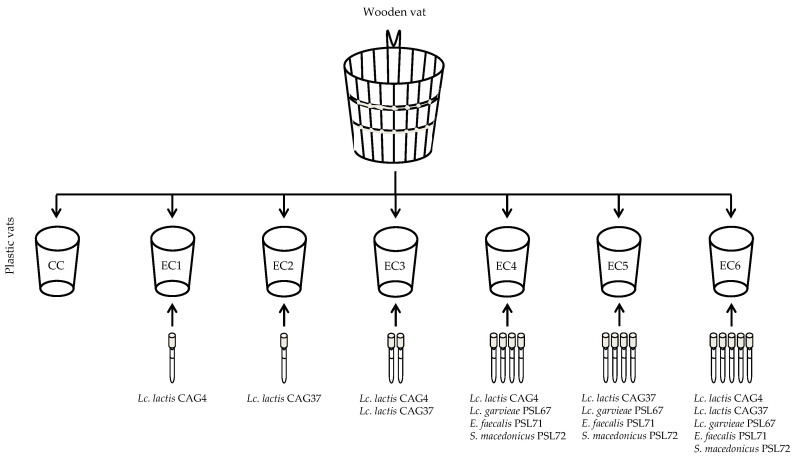
Experimental design of PDO Pecorino Siciliano cheese production performed at the factory-scale level. Adapted from Settanni et al. [16].

Settanni et al. [16] isolated potential starter LAB from acidified PDO Pecorino Siciliano curds, while non-starter LAB was collected from ripened cheeses [6]. Both LAB groups were selected for their main dairy traits: Starter LAB determined the rapid acidification of UHT milk and showed rapid autolysis, while non-starter LAB was chosen for their opposite behavior since a low acidification capacity and slow autolysis are compatible with long ripening times [50]. Sixty-two curd LAB isolates were collected and tested for acidification and autolysis kinetics, but only two strains, both identified as *Lc. lactis* subsp. *lactis*, resulted in rapid acidifiers and highly autolytic. The secondary adjunct culture was composed of three strains (one strain per *Lc. garvieae*, *E. faecalis* and *S. gallolyticus* subsp. *macedonicus*), which were the weakest acidifiers and showed the slowest autolytic activity. These five cultures, individually or in starter/non-starter LAB combination, were applied to produce experimental cheeses in the dairy factory whose cheeses showed the highest levels of spoilage and potentially pathogenic bacteria by Todaro et al. [6]. The bulk milk was collected into the wooden vat daily used by the cheese-maker in order to enrich the milk with the vat biofilms. The entire milk volume was divided into seven aliquots and transferred into plastic vats. One vat was not inoculated with any exogenous LAB culture and represented the control trial, while six vats received LAB inoculums according to the experimental plan (Figure 6). The cheeses were produced applying the traditional PDO Pecorino Siciliano cheese-making protocol, and the ripening lasted five months.

The experimental trials were first followed for their acidification kinetics by monitoring pH and microbiological levels. Both starter strains determined a similar pH decrease in the six inoculated trials, and their pH drop was faster than that registered for the control trial. After 7 days of acidification, the control cheeses showed higher levels of *Pseudomonads* spp. and members of *Enterobacteriaceae* family than cheeses produced with the starter/non-starter LAB inoculums. At the end of the ripening period, prolonged for five months to perform the direct comparison with the cheeses analyzed by Todaro et al. [6], both *Pseudomonads* spp. and members of *Enterobacteriaceae* family were below the detection limit in all inoculated cheeses, while their levels were 3.7 cfu/g (log10) in control cheeses. The species *Lc. lactis* subsp. *lactis* successfully reduced the presence of *Enterobacteriaceae* family members also when added as starter culture during the production of raw ewes’ milk Portuguese Serra de Estrela cheese [72] and different traditional Serbian cheeses [73]. The addition of selected *Lc. lactis* subsp. *lactis* isolated from PDO Pecorino Siciliano curds in milk with microbiological levels close to the limits of the CE Regulation 853 [69] ameliorated the hygienic conditions of the final cheeses. Furthermore, the sensory analysis showed that the cheeses produced with the starter/non-starter LAB inoculums retained a typical sensory profile. Thus, the performances of the selected LAB needed to be tested at a large scale level considering other dairy factories within the consortium for the protection and promotion of PDO Pecorino Siciliano cheese.

## 6. Microbial Stabilization of PDO Pecorino Siciliano Cheese Production

**Figure 7:**
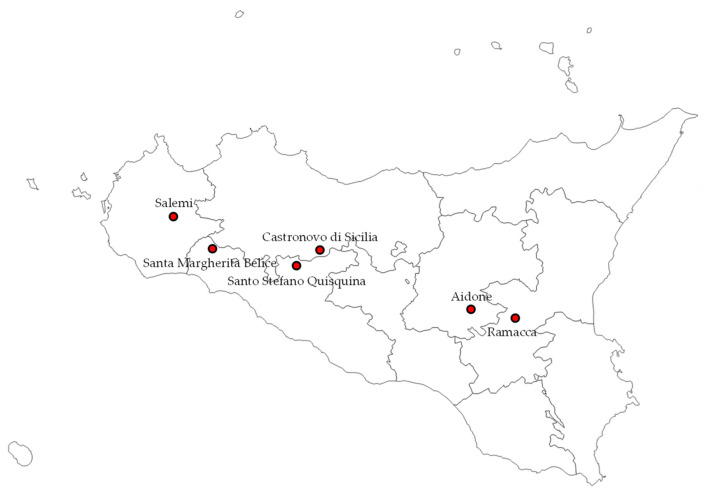
Location of the dairy factories producing PDO Pecorino Siciliano cheese.

The approach of Settanni et al. [16] was followed by Guarcello et al. [74] to extend the results of the selection of the mixed starter (*Lc. lactis* subsp. *lactis*)/non-starter (*E. faecalis*, *Lc. garviae*, *S. gallolyticus* subsp. *macedonicus*) LAB inoculum to six dairy factories located in different areas of Sicily (Figure 7) within five provinces (Agrigento, Catania, Enna, Palermo, and Trapani), all being part of the consortium for PDO Pecorino Siciliano cheese protection and promotion. The application of the selected LAB was performed in an attempt to stabilize the microbiological characteristics of PDO Pecorino Siciliano cheese, preserving its typicality, throughout the regional territory where the disciplinary allows the production.

**Figure 8:**
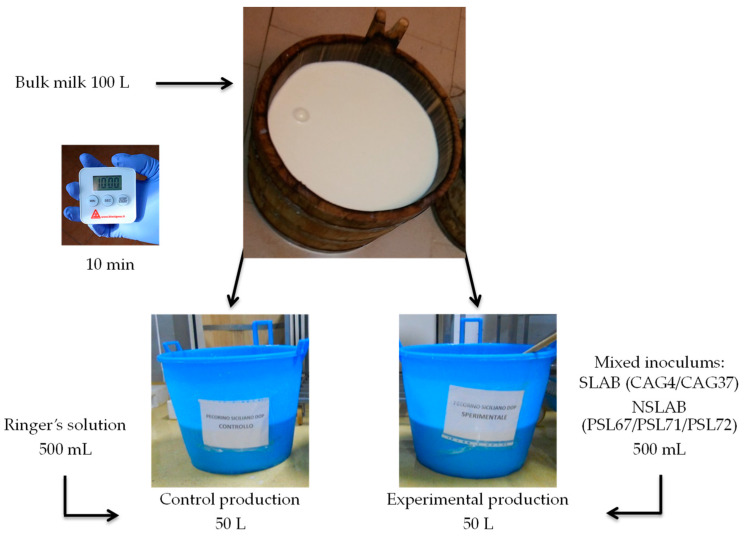
Working plan for PDO Pecorino Siciliano cheese production applied to test the starter/non-starter LAB inoculums at a large scale level.

The production of the cheeses in each factory was performed from the bulk milk after its contact with the wooden vat surface and then transferred into two plastic vats (Figure 8). The control cheeses were processed without LAB inoculums, while the experimental cheeses were inoculated with the mixed starter/non-starter LAB culture. Both cheese productions followed PDO Pecorino Siciliano cheese protocol, and the final cheeses were ripened for five months as for previous investigations.

In general, the level of LAB cocci in the wooden vat biofilms was at higher levels than those of LAB rods. Regarding the bulk milks, they showed similar levels for LAB cocci and total mesophilic counts, thus, LAB dominated the microbial community of all raw milks processed into cheese. The cheese-making parameters were particularly variable among the six dairy factories, but all curds of the trials with selected LAB addition displayed a faster pH drop than control curds. The levels of LAB in ripened inoculated cheeses were higher than those registered in control cheeses. Cheese microbiotas were analyzed, applying a culture-dependent approach exclusively. The authors also performed a polymorphic profile recognition (by randomly amplified polymorphic DNA analysis) of the bacterial isolates from the agar plates at the highest cell suspension dilutions showing the dominance of the inoculated LAB over the vat LAB and those indigenous of milk. Regarding the undesired bacterial groups, coagulase-positive staphylococci were statistically different among the factories, members of the *Enterobacteriaceae* family were at low levels, while pseudomonads were below the detection limit.

**Figure 9:**
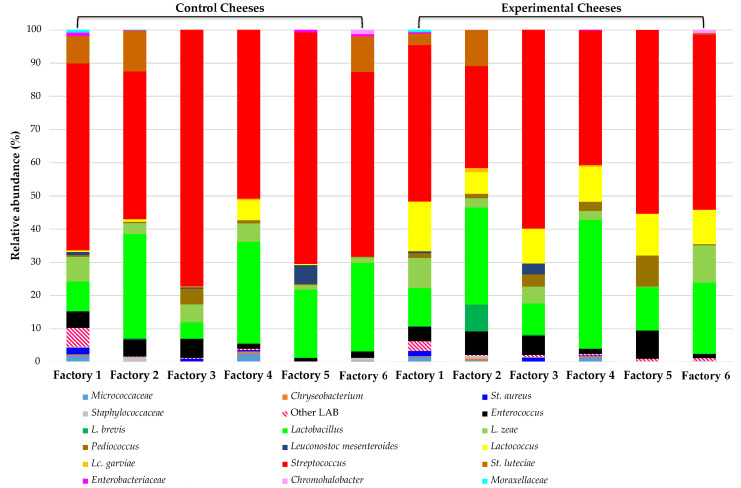
Relative abundances (%) of bacteria identified by MiSeq Illumina in control and experimental PDO Pecorino Siciliano cheeses. Adapted from Gaglio et al. [70].

The microbial stabilization of PDO Pecorino Siciliano cheese production through the addition of the selected starter/non-starter LAB was better investigated by a culture-independent approach based on MiSeq Illumina technology by Gaglio et al. [75]. Cheese microbiota analysis showed that all control cheeses were dominated by streptococci and lactobacilli, while the cheeses processed adding the selected LAB were characterized by dominant levels of streptococci, lactobacilli, and lactococci (Figure 9). In particular, in addition to the LAB species inoculated, this approach also revealed the presence of *Lt. casei* (formerly *Lactobacillus zeae*) in all cheeses, *L. brevis* and *Ln. mesenteroides* only in some factories in both control and inoculated cheeses. The selected LAB culture determined a slight increase in the levels of enterococci.

The presence of *Staphylococcus* spp. in control and experimental cheeses of three factories indicated that even after the addition of selected LAB, some cheeses may be characterized by the presence of pathogenic species and that milk hygiene is important for the safety of the final cheeses. Other minor groups including *Chryseobacterium*, *Chromohalobacter, Micrococcaceae,* and *Moraxellaceae* were also detected.

The final cheeses were analyzed for the chemical composition. The addition of the selected LAB did not affect cheese fatty acids (FA), mainly represented by saturated fatty acids like palmitic and myristic acids. The ratio between saturated FA and unsaturated FA, in particular oleic acid, for control and selected LAB added cheeses were almost superimposable (2.31 and 2.29, respectively). Regarding the volatile organic compound composition of the cheeses, their profiles were composed of alkanes, aldehydes, terpenes, esters, acids, ketones, and benzene. The cheeses inoculated with the selected LAB were different from those of the control cheeses only for butanoic acid, but the factories where the cheeses were produced impacted differently the presence of hexane and hexanoic acid ethyl ester that was generated at higher concentrations from the control cheeses [75]. The addition of the starter/non-starter LAB addition impacted the final pH of the ripened cheeses that was 0.1 points lower than the average value registered for the control cheeses. Specifically, the final pH of control cheeses was 5.76, while that of LAB added cheeses was 5.66. This finding could be of relevance in the practice of producing cheeses from raw milk. Even though 0.1 points pH does not strongly impact the acidity perception by consumers, a lower pH of the curd due to the addition of the starter culture rather than non-starter LAB exerts a stressing effect on the undesired microorganisms present in raw milk retarding or inhibiting their development. Some differences regarding cheese yield, water activity (a_w_), ash content, and salt percentage were registered among factories. In particular, cheese yield % and a_w_ was higher for the control cheeses, while ash % on dry matter and salt % were higher in the cheeses added with the selected LAB [74].

The same cheeses were evaluated by 10 expert panelists who scored 21 descriptive attributes, including aroma, taste, surface structure, and texture [74]. The sensory tests evidenced how the addition of LAB influenced color, eye formation, uniformity of structure, and unpleasant taste. The panelists detected fewer eyes in the cheeses produced by adding the selected LAB while a higher presence of unpleasant taste was scored for the control cheeses. Furthermore, a triangle test indicated the highest preference for the cheeses produced with the addition of the LAB inoculums. Although the selected LAB determined a lowering of pH, they did not generate an overproduction of organic acids excluding that they negatively impacted the overall assessment of the cheeses.

## 7. Conclusions and Future Perspectives

### Future Perspectives

Cheese is one of the foods that are undergoing a negative impact among consumers due to their fat content and low presence of active compounds. However, very recently, cheeses are being rediscovered as foods with functional properties, in particular, when ingredients with bioactive compounds are added. From this perspective, the current research is focusing on the incorporation of several ingredients, mainly by-products rich in polyphenols and other antioxidant molecules [76]. Our research group is preparing works on the incorporation of grape pomace wastes and aromatic herbs in Pecorino Siciliano cheeses for which LAB resistant to plant polyphenols have been selected ad hoc [77,78,79]. Furthermore, Pecorino Siciliano cheese was evaluated as a promising matrix to deliver probiotic cultures [80], opening new routes for production and commercialization of this traditional cheese.

## 8. Conclusions 

The quality of cheese productions carried out without the addition of starter cultures is quite unpredictable. In order to stabilize the characteristics of these cheeses and to preserve the typical organoleptic notes avoiding the flattening of the sensory profile of the final products, the application of commercial starter cultures is not advisable [81]. The development of starter and secondary adjunct (non-starter) cultures from autochthonous LAB has been reported as a winning strategy to maintain cheese typicality. Autochthonous LAB are those isolated from the production environment, associated to the local raw materials, and adapted to the traditional process technology [7]. The studies conducted on PDO Pecorino Siciliano cheese showed that the wooden vats used for milk collection and curdling are a primary source of useful LAB, starter cultures selected from autochthonous populations safeguard the typicality of the cheese with little modifications of the traditional manufacture technology, their application in combination with non-starter LAB determines their dominance over milk LAB and controls the development of spoilage and potential pathogenic species. These investigations also indicated that the final cheese safety does not depend exclusively on raw milk, but it is rather a consequence of several technological and microbiological factors occurring during production. Specifically, the addition of the selected mixed culture composed of *Lc. lactis* subsp. *lactis*, *E. faecalis*, *Lc. garviae*, and *S. gallolyticus* subsp. *macedonicus* at a large scale level among several dairy factories gathered into PDO Pecorino Siciliano cheese consortium was able to determine rapid curd acidification and to activate mechanisms of antagonism for the control of undesired microorganisms. This strategy provided useful evidence to preserve the identity of typical cheeses and to improve food security. However, the cooking step undoubtedly represents a key factor for the final safety of raw milk cheeses. From this perspective, the possible interaction among the cooking process, starter selection, and reduction of pathogenic and spoilage bacteria still needs to be investigated for PDO Pecorino Siciliano cheese.

## Data Availability

All data included in this study are available upon request by contacting the corresponding author.
